# Supplementary material for: Interleaved Electroactive Molecules into LDH Working on Both Electrodes of an Aqueous Battery-Type Device
Source: Molecules. 2023 Jan 19;28(3):1006. doi: 10.3390/molecules28031006 (PMC9920818; doi:10.3390/molecules28031006)
Supplement: Supplementary file 1 [file molecules-28-01006-s001.zip › molecules-2134705-supplementary.pdf]

Supplementary information

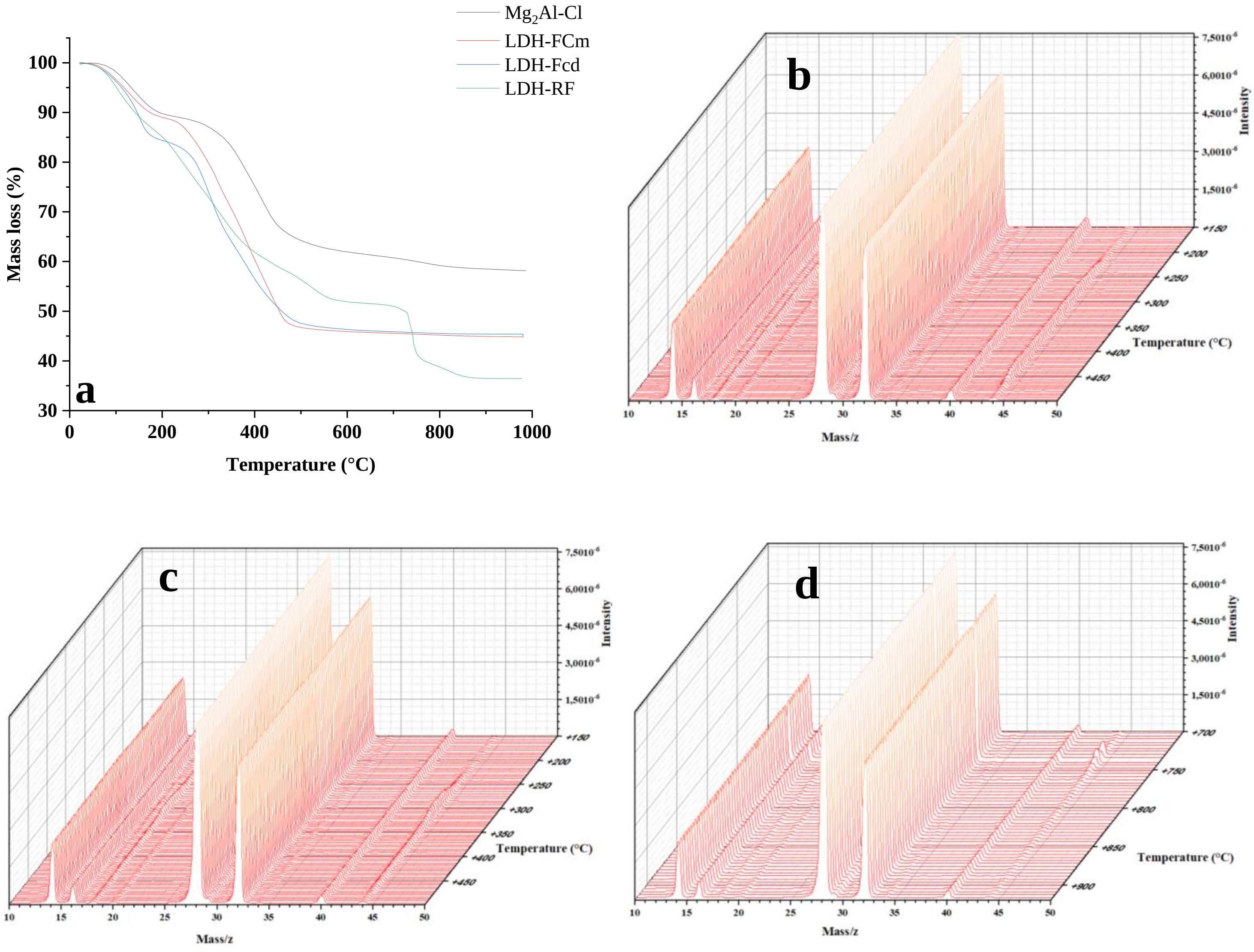

Figure S1. (a) TGA of LDH-Cl, LDH-RF, LDH-FCm and LDH-FCd , and stacked Mass Spectrographs as a function of the temperature for (b) LDH-RF , (c) LDH-FCm, and (d) LDH-FCd

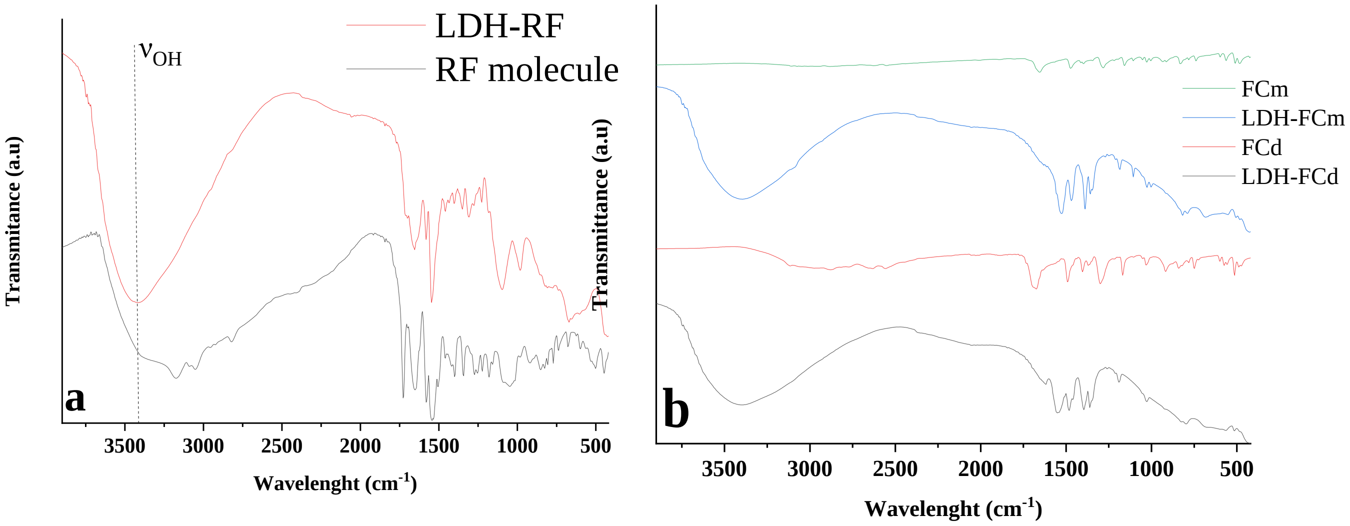

Figure S2. Infrared spectrum of (a) RF and LDH-RF and (b) FCm, LDH-FCm, FCd, LDH-FCd

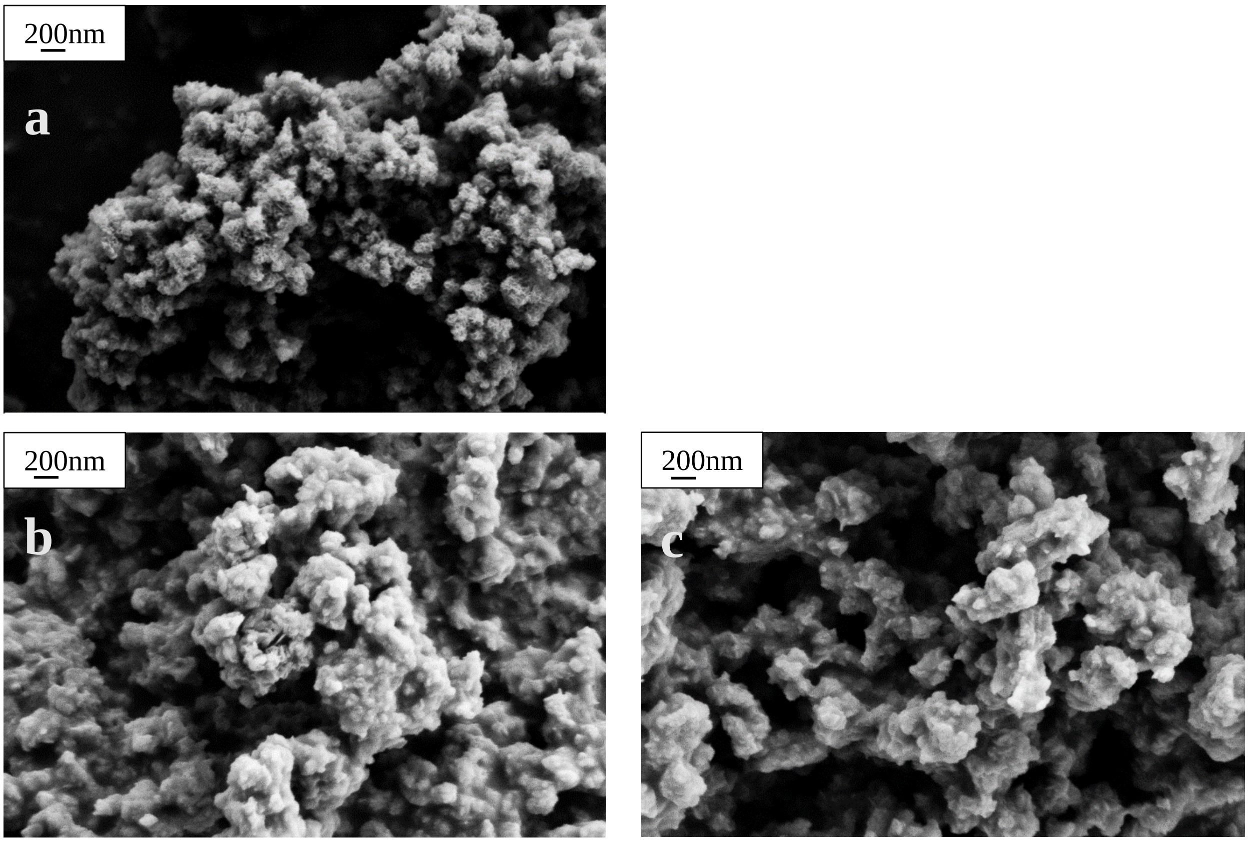

Figure S3. SEM images of (a) LDH-RF, (b) LDH-FCm and (c) LDH-FCd

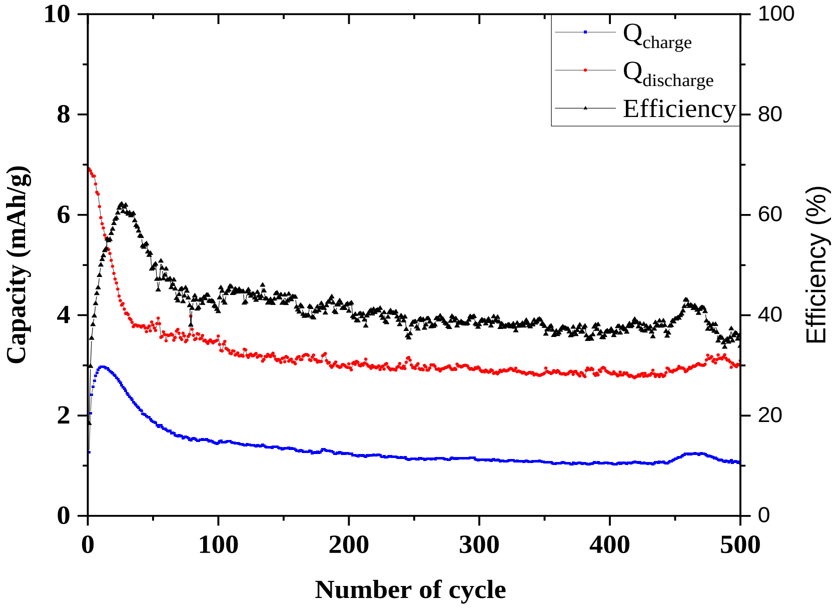

Figure S4. Capacity of RF in sodium acetate 1M at 10mV/s

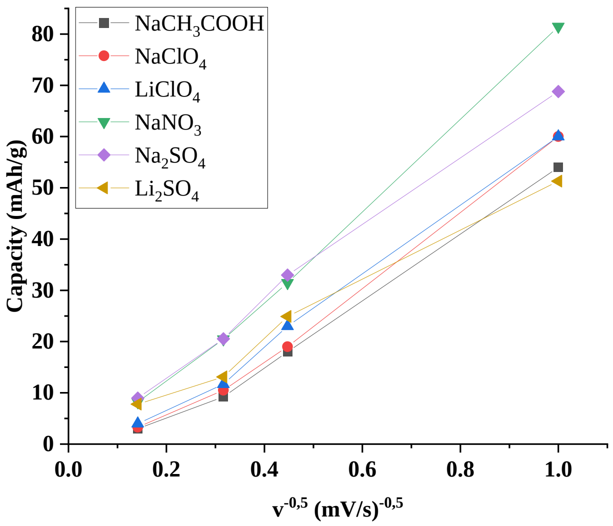

Figure S5. Evolution of capacity depending on scan rate and on the nature of the electrolyte salt.

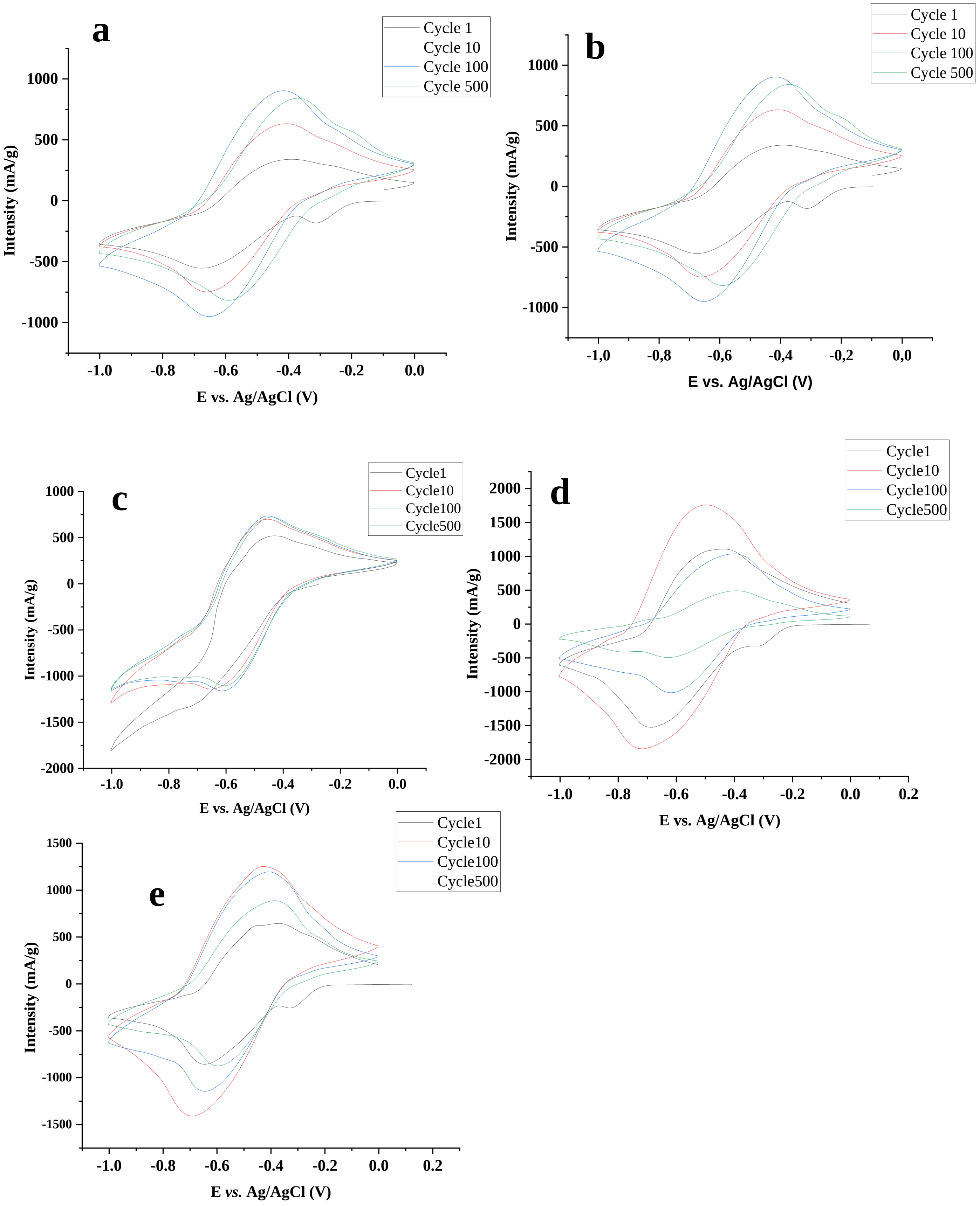

Figure S6. CV experiments of LDH-RF at 10mV/s in a 1M solution of (a) $NaClO_4$, (b) $LiClO_4$, (c) $NaNO_3$, (d) Na_2SO_4 and (e) Li_2SO_4

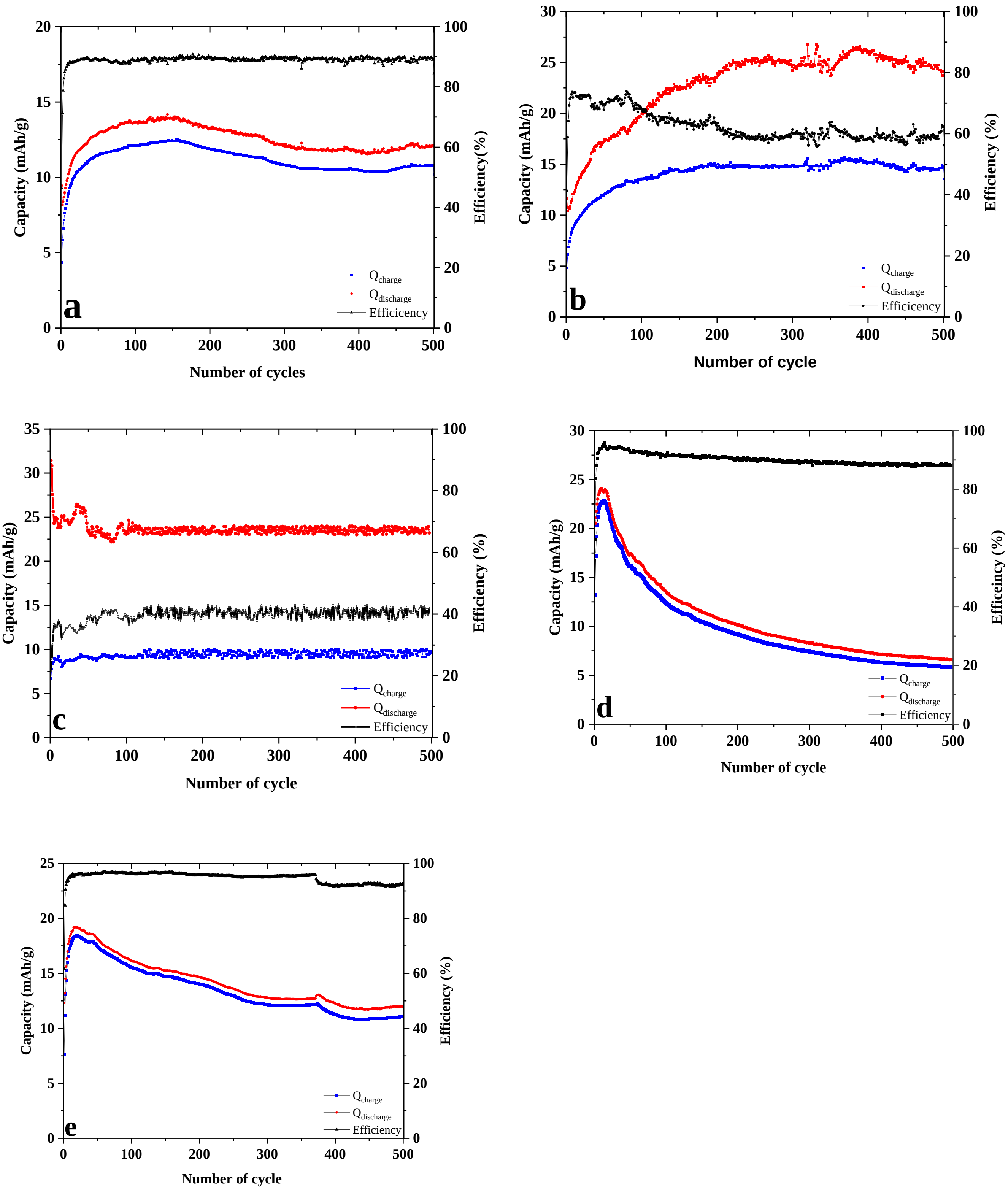

Figure S7. Capacity and efficiency of LDH-RF obtained at 10mV/s in a 1M solution of (a) $NaClO_4$, (b) $LiClO_4$, (c) $NaNO_3$, (d) Na_2SO_4 and (e) Li_2SO_4

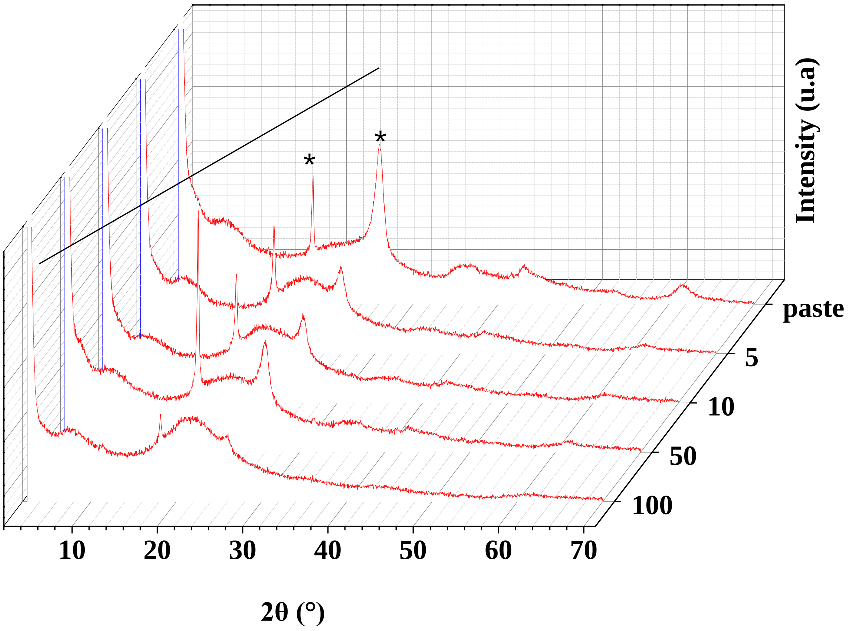

Figure S8. XRD of LDH-RF after 5th, 10th, 50th and 100th cycle in sodium acetate at 10mV/s

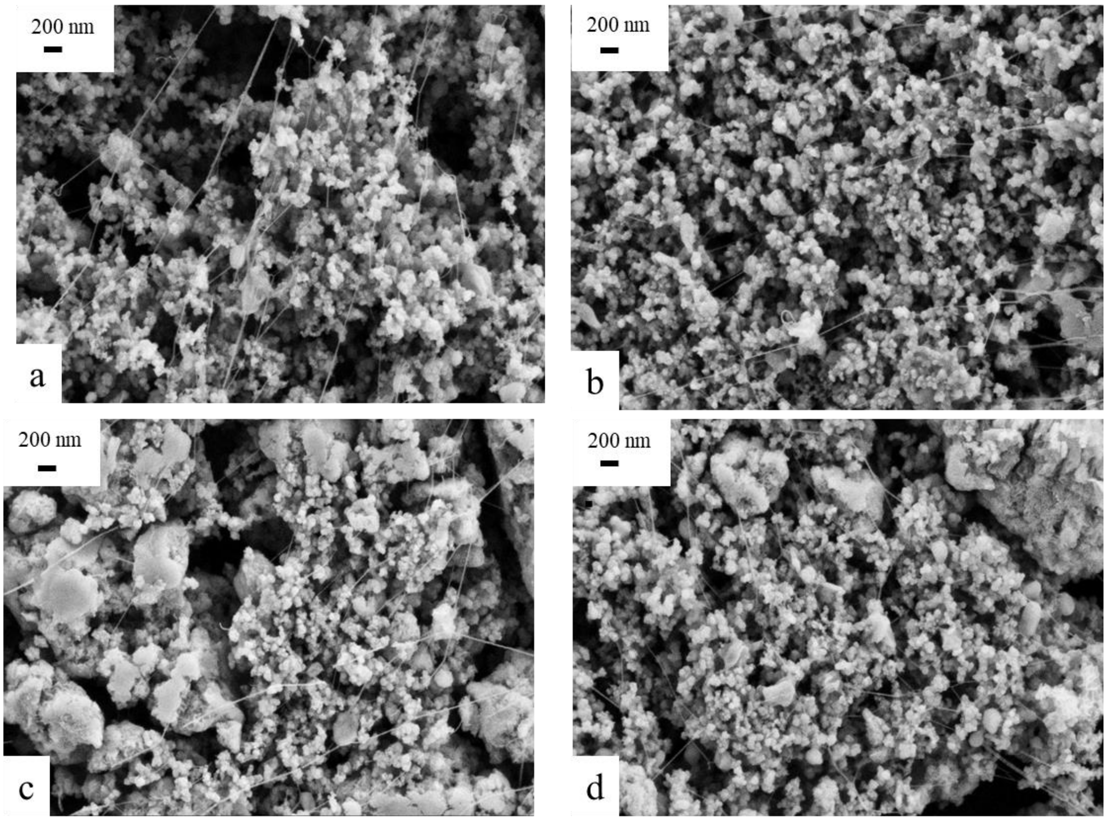

Figure S9. SEM images of LDH-RF paste (a) and post-mortem after 1st (b), 5th (c), 50th (d) cycle in sodium acetate at 10mV/s

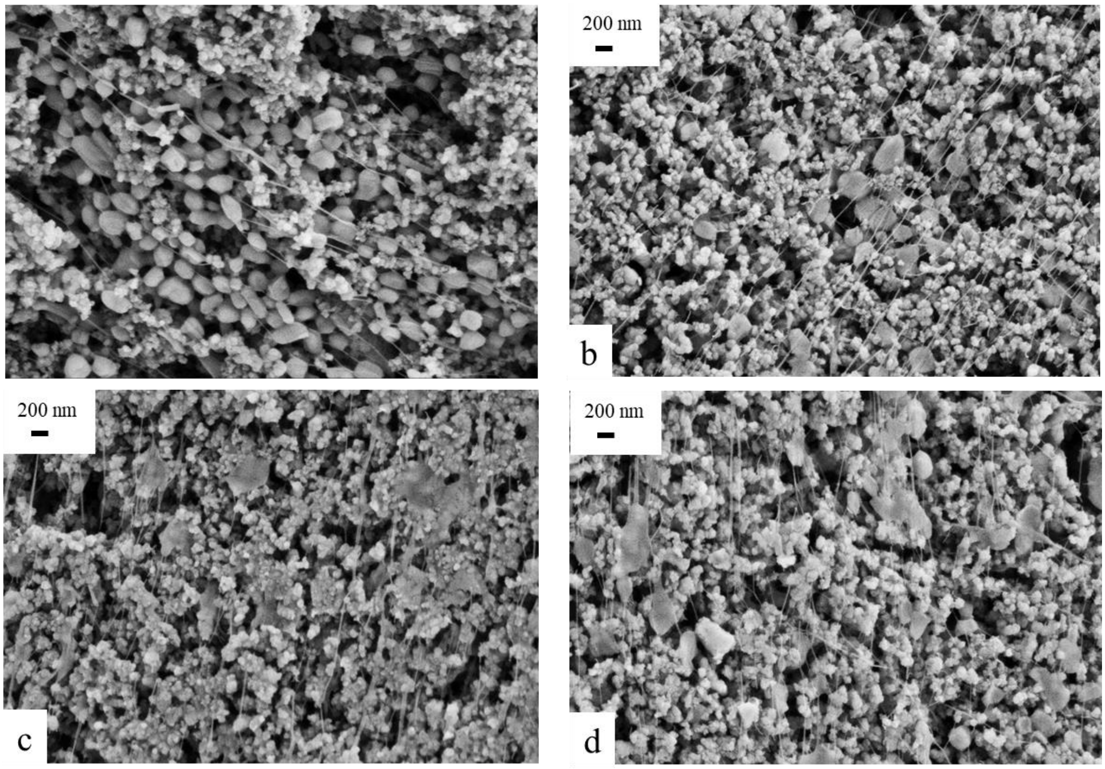

Figure S10. SEM images of LDH-FCm paste (a) and post-mortem after 1st (b), 5th (c), 50th (d) cycle in sodium acetate at 10mV/s

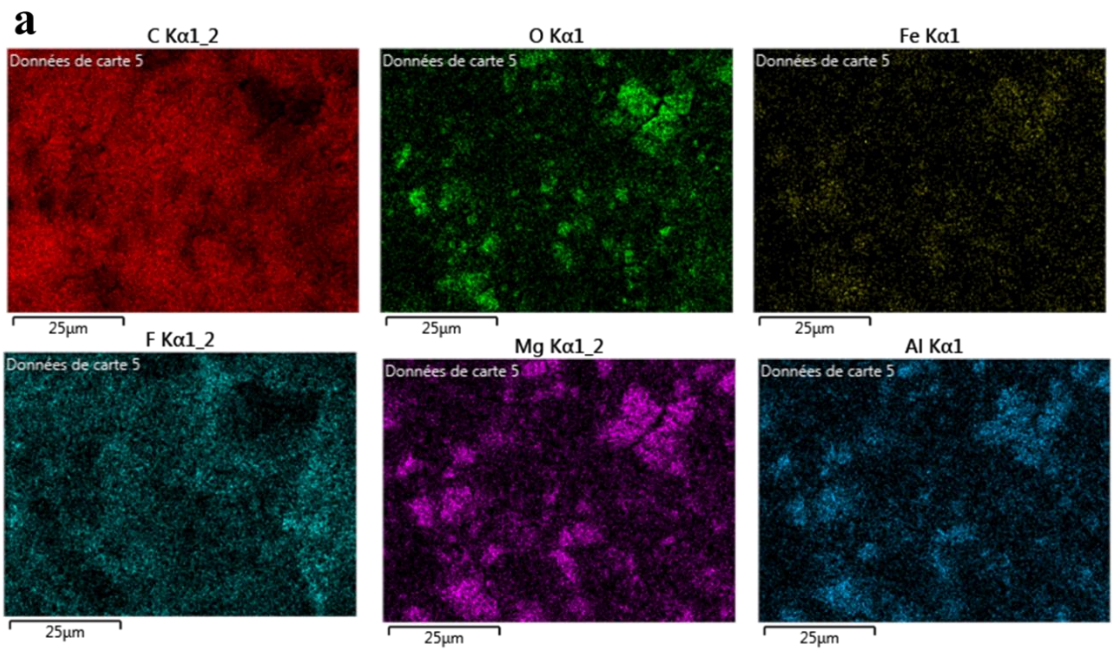

b

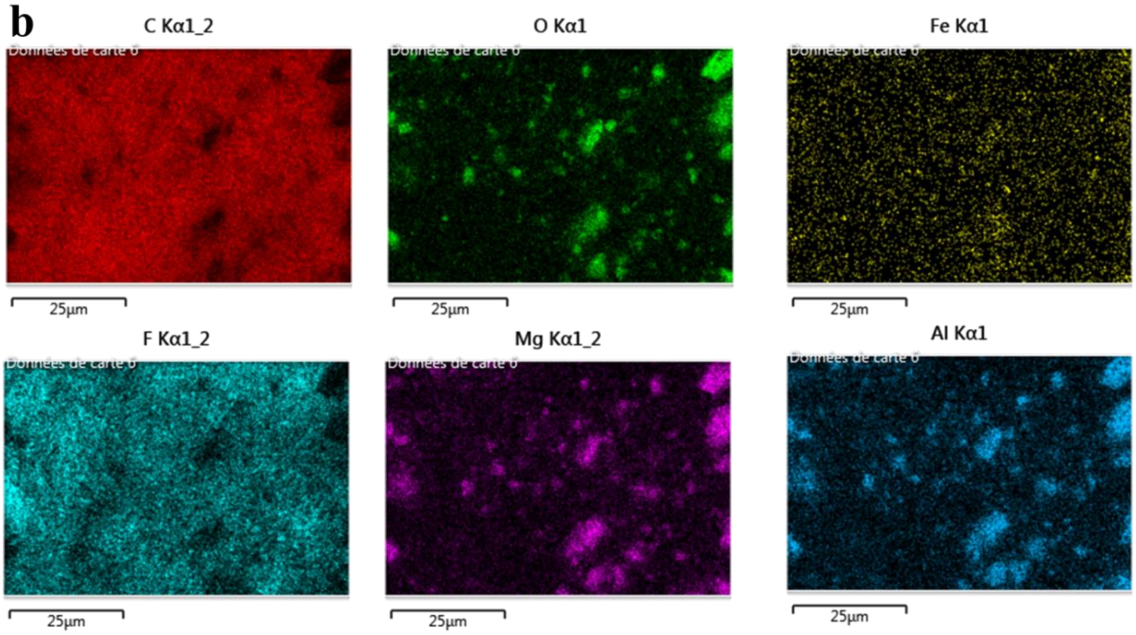

C Kα1_2
O Kα1
Fe Kα1
Données de carte 6
25µm
F Kα1_2
Mg Kα1_2
Al Kα1

c

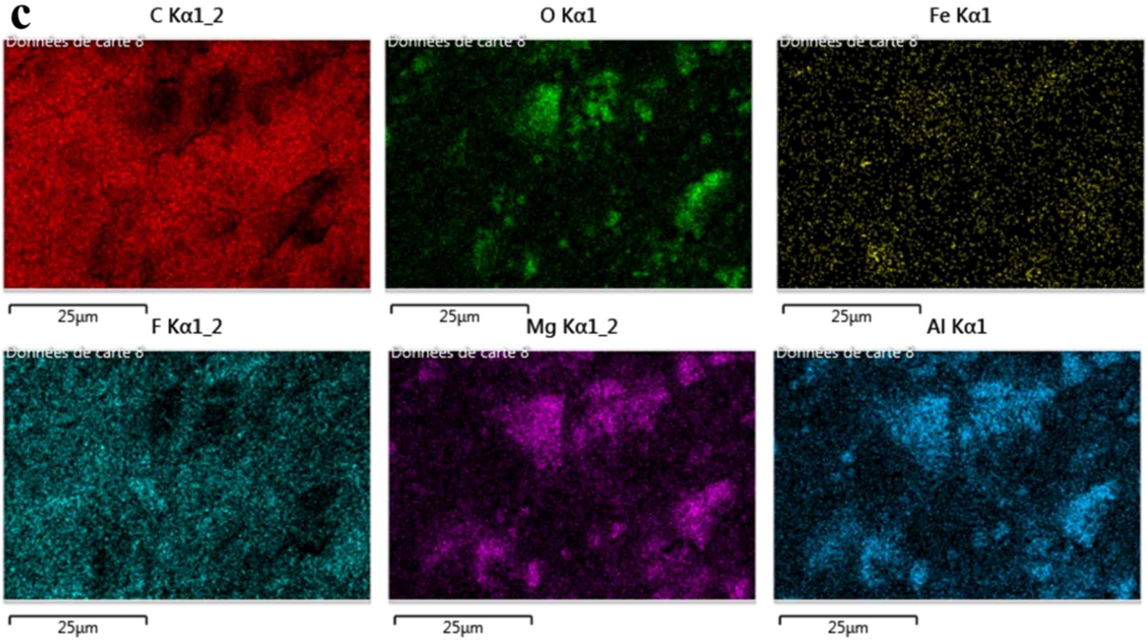

C Kα1_2
O Kα1
Fe Kα1
Données de carte 8
25µm
F Kα1_2
Mg Kα1_2
Al Kα1

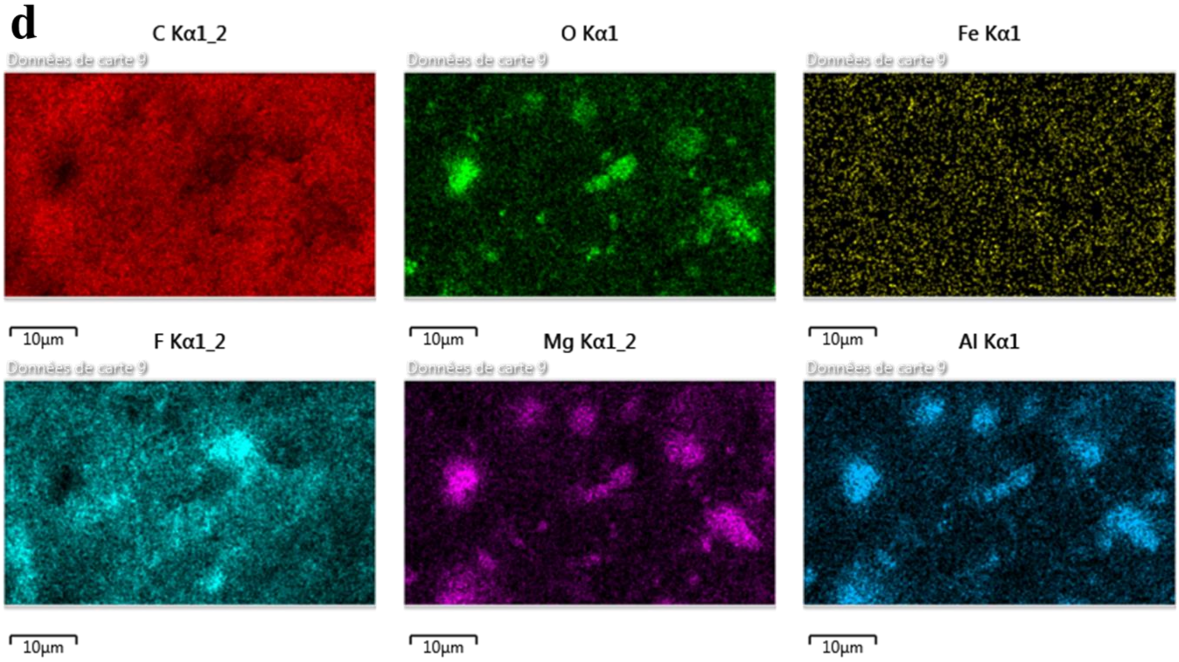

Figure S11. EDX mapping of (a) LDH-Fcm raw paste, LDH-FCm paste after (b)1st cycle, (c) 5th cycle and (d) 50th in sodium acetate 1M at 10mV/s

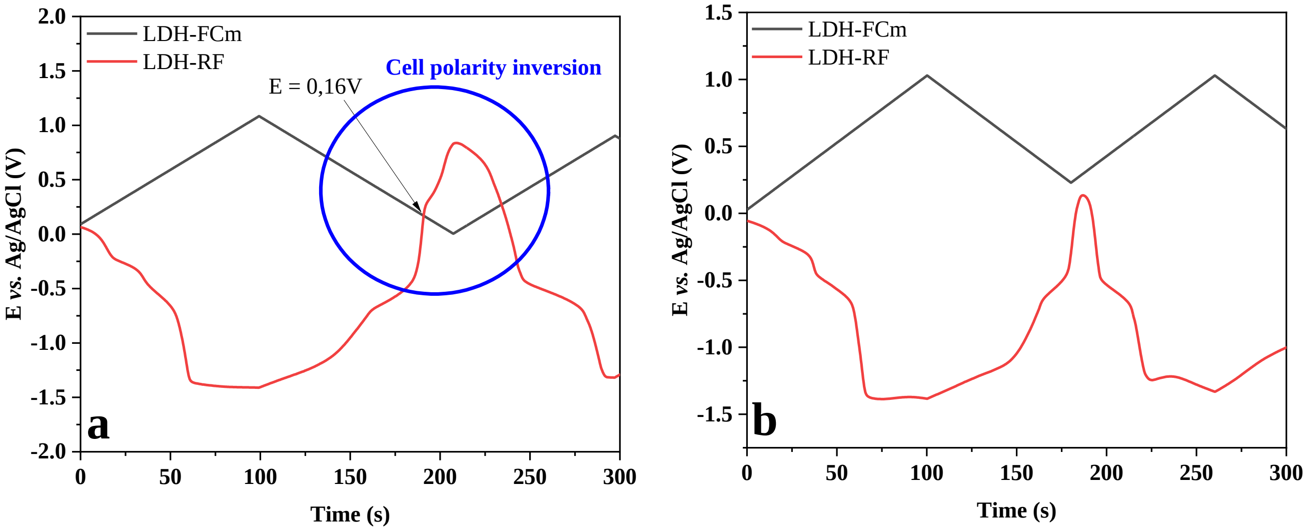

Figure S12. (a) Potential evolution of LDH-Fcm and LDH-RF electrodes in a complete cell with [0;1] V potential range imposed for LDH-Fcm in 1M sodium acetate at 10mV/s and (b) Potential evolution of LDH-Fcm and LDH-RF electrodes in a complete cell with [0,2;1] V potential range imposed for LDH-Fcm in 1M sodium acetate at 10mV/s

Table S1: Results of CV experiments of LDH-RF in different electrolyte at 1mV/s

Electrolyte	*Eox (V)*	*Ered (V)*	*Qc_{1st} cycle*	*Qd_{1st} cycle*	*Efficiency (%)*	*Qc_{25th} cycle*	*Qd_{25th} cycle*	*Efficiency (%)*
$NaCH_3COOH$	-0.49	-0.63	10.0	30.4	17	21.0	73.3	29
$NaClO_4$	-0.38	-0.60	16.4	70.0	23	19.0	55.6	34
$LiClO_4$	-0.32	-0.71	17.1	54.0	32	25.2	97.8	26
$NaNO_3$	-0.42	-0.58	8.0	81.4	10	/	/	/
Na_2SO_4	-0.57	-0.74	25.9	68.8	37	/	/	/
Li_2SO_4	-0.39	-0.74	28.1	51.2	55	10.3	24.7	41

Table S2: Results of CV experiments of LDH-FCd in different electrolyte at 1mV/s

Electrolyte	*Eox (V)*	*Ered (V)*	*Qc_{1st} cycle*	*Qd_{1st} cycle*	*Efficiency (%)*	*Qc_{5th} cycle*	*Qd_{5th} cycle*	*Efficiency (%)*
$NaCH_3COOH$	0.50	0.38	5.3	2.3	43	1.1	0.8	73
$NaClO_4$	0.55	0.42	9.0	2.9	32	4.0	3.4	86
$LiClO_4$	0.61	0.40	6.4	2.7	40	6.5	4.9	75
Na_2SO_4	0.65	0.51	14.3	2.5	18	4.8	2.1	46
Li_2SO_4	0.59	0.51	9.5	1.1	12	0.8	0.2	30

Table S3: Results of CV experiments of LDH-FCm in different electrolyte salts at 1mV/s

Electrolyte	*Eox (V)*	*Ered (V)*	*Qc_{1st} cycle*	*Qd_{1st} cycle*	*Efficiency (%)*	*Qc_{5th} cycle*	*Qd_{5th} cycle*	*Efficiency (%)*
$NaCH_3COOH$	0.38	0.29	59.9	38.8	65	6.2	0.9	15
$NaClO_4$	0.52	0.32	60.1	28.4	47	19.0	11.2	59
$LiClO_4$	0.62	0.22	60.6	24.0	40	25.5	18.7	72
Na_2SO_4	0.53	0.27	59.3	2.8	4.7	5.6	0.6	10
Li_2SO_4	0.48	0.28	57.6	0.5	0.8	4.0	0.4	10
